# A rapid *in vivo* screen for pancreatic ductal adenocarcinoma therapeutics

**DOI:** 10.1242/dmm.020933

**Published:** 2015-10-01

**Authors:** Ozhan Ocal, Victor Pashkov, Rahul K. Kollipara, Yalda Zolghadri, Victoria H. Cruz, Michael A. Hale, Blake R. Heath, Alex B. Artyukhin, Alana L. Christie, Pantelis Tsoulfas, James B. Lorens, Galvin H. Swift, Rolf A. Brekken, Thomas M. Wilkie

**Affiliations:** 1 Department of Pharmacology, UT Southwestern Medical Center, Dallas, TX 75390, USA; 2 Department of Neuroscience, UT Southwestern Medical Center, Dallas, TX 75390, USA; 3 Department of Basic Sciences, School of Veterinary Medicine, Shiraz University, Shiraz, Iran; 4 Department of Surgery andHamon Center for Therapeutic Oncology Research, UT Southwestern Medical Center, Dallas, TX 75390, USA; 5 Department of Molecular Biology, UT Southwestern Medical Center, Dallas, TX 75390, USA; 6 Department of Physiology and Biophysics, Virginia Commonwealth University, Richmond, VA 23298, USA; 7 Simmons Cancer Center, UT Southwestern Medical Center, Dallas, TX 75390, USA; 8 Department of Neurological Surgery, University of Miami School of Medicine, Miami, FL 33136, USA; 9 Department of Biomedicine, University of Bergen, N-5009 Bergen, Norway

**Keywords:** *Rgs16::GFP* reporter, Kras, Rapid *in vivo* screen, Pancreatic cancer combination therapy, Gas6, Axl, Warfarin, Gemcitabine, Abraxane

## Abstract

Pancreatic ductal adenocarcinoma (PDA) is the fourth leading cause of cancer-related deaths in the United States, and is projected to be second by 2025. It has the worst survival rate among all major cancers. Two pressing needs for extending life expectancy of affected individuals are the development of new approaches to identify improved therapeutics, addressed herein, and the identification of early markers. PDA advances through a complex series of intercellular and physiological interactions that drive cancer progression in response to organ stress, organ failure, malnutrition, and infiltrating immune and stromal cells. Candidate drugs identified in organ culture or cell-based screens must be validated in preclinical models such as *KIC* (*p48^Cre^;LSL-Kras^G12D^;Cdkn2a^f/f^*) mice, a genetically engineered model of PDA in which large aggressive tumors develop by 4 weeks of age. We report a rapid, systematic and robust *in vivo* screen for effective drug combinations to treat Kras-dependent PDA. Kras mutations occur early in tumor progression in over 90% of human PDA cases. Protein kinase and G-protein coupled receptor (GPCR) signaling activates Kras. Regulators of G-protein signaling (RGS) proteins are coincidence detectors that can be induced by multiple inputs to feedback-regulate GPCR signaling. We crossed *Rgs16::GFP* bacterial artificial chromosome (BAC) transgenic mice with *KIC* mice and show that the *Rgs16::GFP* transgene is a Kras^G12D^-dependent marker of all stages of PDA, and increases proportionally to tumor burden in *KIC* mice. RNA sequencing (RNA-Seq) analysis of cultured primary PDA cells reveals characteristics of embryonic progenitors of pancreatic ducts and endocrine cells, and extraordinarily high expression of the receptor tyrosine kinase Axl, an emerging cancer drug target. In proof-of-principle drug screens, we find that weanling *KIC* mice with PDA treated for 2 weeks with gemcitabine (with or without Abraxane) plus inhibitors of Axl signaling (warfarin and BGB324) have fewer tumor initiation sites and reduced tumor size compared with the standard-of-care treatment. Rgs16::GFP is therefore an *in vivo* reporter of PDA progression and sensitivity to new chemotherapeutic drug regimens such as Axl-targeted agents. This screening strategy can potentially be applied to identify improved therapeutics for other cancers.

## Abstract (precis)

**Editor's choice - Drug Discovery Collection:** The study describes a rapid *in vivo* screen for novel strategies to treat pancreatic ductal adenocarcinoma (PDA).

## INTRODUCTION

Pancreatic ductal adenocarcinoma (PDA) is the fourth leading cause of cancer-related deaths but is predicted to become more common owing to its association with smoking, diet, obesity and type 2 diabetes (Pannala et al., 2008; Rahib et al., 2014; Siegel et al., 2015). Three major classifications of pancreatic precancerous lesions are associated with progression to PDA: PanIN (pancreatic intraepithelial neoplasia), IPMN (intraductal papillary mucinous neoplasm) and MCN (mucinous cystic neoplasm) (Distler et al., 2014). Precancerous lesions can be common in the elderly or obese. For example, early PanINs were found in 65% of obese patients, and their presence was associated with intravisceral fat, and pancreatic intralobular fibrosis and fat (Rebours et al., 2015). IPMNs are the next most common pancreatic precancerous lesion associated with PDA (Maitra et al., 2005). They are found in the pancreatic main and branching ducts. MCNs occur predominantly in females, predominantly in the peripheral pancreas (Thompson et al., 1999).

Recent mathematical predictions attribute spontaneous mutations during cell division as initiators of PDA, making early detection and effective therapy the only two elements determining survival (Tomasetti and Vogelstein, 2015). Unfortunately, PDA symptoms present late in disease progression and, other than surgical resection, limited progress has been made in developing effective treatments after gemcitabine was introduced as a first-line therapy for advanced PDA (Burris et al., 1997). Gemcitabine treatment alone or after resection is marginally effective in prolonging survival. One of the two predominant therapeutic regimens is gemcitabine combined with nab-paclitaxel (Abraxane), which was shown to increase survival to 8.5 months, compared with 6.7 months for patients who received gemcitabine alone (Von Hoff et al., 2013). In a follow-up study, 3% of patients in the gemcitabine plus nab-paclitaxel group were still alive after 42 months of treatment compared with none in the gemcitabine only group (Goldstein et al., 2015). The primary mechanism of function of paclitaxel is interference with microtubule depolymerization leading to mitotic failure (Schiff et al., 1979, 1980). Nab-paclitaxel has been shown to provide better tolerance and absorption than paclitaxel. In addition, nab-paclitaxel augments gemcitabine efficacy by reducing the level of its metabolizing enzyme, cytidine deaminase (Ibrahim et al., 2002; Frese et al., 2012). However, tumors are often resistant to this combination (Neesse et al., 2014). The other common drug treatment, FOLFIRINOX, consisting of four different chemotherapy agents, is more effective but less well-tolerated (Becker et al., 2014; Moorcraft et al., 2014; Haeno et al. 2012). Therefore, there is a need for a systematic and robust *in vivo* screen that can accelerate the pace of discovery of improved PDA therapeutics.

**TRANSLATIONAL IMPACT**

**Clinical issue**

Pancreatic ductal adenocarcinoma (PDA) is the fourth leading cause of cancer-related US deaths, and is projected to be the second leading cause by 2025 because of its association with smoking, obesity and type 2 diabetes. PDA has the worst survival rate of any major cancer so far. The current standard-of-care provides only modest therapeutic gains. The two most desperately needed advances for extending life expectancy of individuals with PDA are improved therapeutics and the identification of early markers. PDA advances through a complex series of intercellular and physiological interactions that drive cancer proliferation in response to organ stress, and infiltrating immune and stromal cells, causing organ failure and subsequent malnutrition. *Kras* mutations occur early in tumor progression in over 90% of human PDA. However, Kras is refractory to direct inhibitors.

**Results**

In this study, the authors report a rapid, systematic and robust *in vivo* screen for effective drug combinations to treat Kras-dependent PDA. Protein kinase and G-protein coupled receptor (GPCR) signaling activates Kras. Regulators of G-protein signaling (RGS) proteins are coincidence detectors that can be induced by multiple inputs to feedback-regulate GPCR signaling. The *Rgs16::GFP* transgene is a *Kras^G12D^*-dependent marker of all stages of PDA neoplasia and its expression increases proportionally to tumor burden in *KIC* mice – a genetically engineered mouse model of PDA. RNA sequencing analysis of cultured primary PDA cells shows characteristics of embryonic progenitors of pancreatic ducts and endocrine cells, and extraordinarily high expression of the receptor tyrosine kinase Axl, an emerging cancer drug target. In proof-of-principle drug screens, the authors find that PDA weanling mice treated for 2 weeks with gemcitabine plus Abraxane and inhibitors of Axl signaling (warfarin or BGB324) have fewer tumor initiation sites and reduced tumor size compared with *KIC* mice treated with standard-of-care treatments (either gemcitabine alone or gemcitabine plus Abraxane).

**Implications and future directions**

Candidate anti-cancer drugs identified in organ culture or cell-based screens must be validated for efficacy in preclinical models such as *KIC* mice. *Rgs16::GFP* is a robust and faithful *in vivo* reporter of PDA progression and sensitivity to new chemotherapeutic drug regimens, including Axl-targeted agents. This rapid *in vivo* screening strategy could potentially be applied to identify improved therapeutics for many other cancers.

PDA initiates as ductal neoplasia, derived from any of three pancreatic adult cell types – ductal progenitor cells, centroacinar cells, or acinar cells that have undergone acinar-to-ductal metaplasia (ADM) (Bonner-Weir et al., 2004; Rovira et al., 2010; von Figura et al., 2014). In humans, activated Kras and inactivated Cdkn2a are the earliest and most common genetic mutations identified in disease progression (Hezel et al., 2006; Iacobuzio-Donahue et al., 2012). Genetically engineered mouse models (GEMMs) based on these mutations have been developed to investigate PDA initiation and propagation. In the present study, we use *KC* (*p48^Cre^;LSL-Kras^G12D^*) and *KIC* (*p48^Cre^;LSL-Kras^G12D^;Cdkn2a^f/f^*) mice. Both lines form tumors because they express activated Kras^G12D^ (*KIC* also has inactivation of the tumor suppressor Cdkn2a) in all three pancreatic lineages – ducts, acinar and endocrine cells – under control of the *p48* (*Ptf1a*) promoter. By contrast, *IC* mice (*p48^Cre^;Cdkn2a^f/f^*) never form tumors (Aguirre et al., 2003). *KIC* mice are an excellent GEMM for PDA therapeutic screens because neoplasia develops early, between 2 to 3 weeks of age, and large aggressive tumors develop in all mice by 4 weeks of age (Aguirre et al., 2003).

PDA is the most frequent major cancer harboring Ras mutations (e.g. Kras^G12D^) (Pylayeva-Gupta et al., 2011). Kras mutations are found in over 90% of human PDA cases (Iacobuzio-Donahue et al., 2012). Kras^G12D^ expression is necessary but not sufficient to initiate neoplasia; GTP binding is required to activate Kras^G12D^ (Huang et al., 2014). Ras guanine nucleotide exchange factors (Ras-GEFs) catalyze GDP dissociation, and subsequent GTP binding to Ras (Jeng et al., 2012). Protein kinase and G-protein coupled receptor (GPCR) signaling can stimulate Ras-GEFs to promote Kras^G12D^-dependent neoplasia (van Blesen et al., 1995; Kahn, 2014). Regulators of G-protein signaling (RGS) proteins are GTPase activating proteins (GAPs) for the Gi- and Gq-alpha subunits of heterotrimeric G proteins (Berman et al., 1996). Interestingly, RGS-resistant mutations in Gα_q_ (and Gα_s_) were found in cells isolated from pancreatic cysts (Wu et al., 2011). RGS proteins are coincidence detectors that can be induced by and integrate multiple inputs to feedback-regulate the GPCR arm of the pathway, by virtue of their Gα-GAP activity (Ross and Wilkie, 2000; Huang et al., 2006; Villasenor et al., 2010; Pashkov et al., 2011). The induction of RGS proteins can therefore be monitored to report hyperactivated Ras signaling (Dohlman et al., 1996; Dignard et al., 2008). Because Ras remains an elusive drug target (Stephen et al., 2014), we developed an *in vivo* screen for PDA therapeutics that is responsive to Kras signaling.

We previously described expression of an *Rgs16::GFP* bacterial artificial chromosome (BAC) transgene during embryonic and postnatal pancreas development in pancreatic progenitors, endocrine cells and duct cells (Villasenor et al., 2010). GFP was expressed in ducts and islet β-cells during neonatal pancreas development but was not detected in euglycemic adult mice. Rgs16::GFP was reactivated, first in ducts, then islet β-cells, under conditions of chronic insulin demand or hyperglycemia in mouse models of type 1 and type 2 diabetes, and during gestation. In humans, Rgs16 expression was observed in ducts of pancreatic cancer patients prior to detectable metastasis (Kim et al., 2010). Chronic stress might induce Rgs16 in progenitor cells within the pancreatic ductal epithelium (Bonner-Weir et al., 2004; Villasenor et al., 2010). To test whether Rgs16 is an early marker of PDA, we crossed the *Rgs16::GFP* transgene into *KIC* mice. Here, we show that the *Rgs16::GFP* transgene is a Kras^G12D^-dependent marker of all stages of neoplasia in *KIC* mice – IPMN, PanIN and PDA (Hruban et al., 2000; Maitra et al., 2005). The distribution and intensity of Rgs16::GFP expression is proportional to and coincident with tumor burden.

In a proof-of-principle for drug screens, we find that weanling *KIC* mice with PDA treated with the standard-of-care combination of gemcitabine and nab-paclitaxel (Abraxane) (Masellis et al., 2009; Frese et al., 2012; Von Hoff et al., 2013; Neesse et al., 2014) for 2 weeks have significantly lower *Rgs16::GFP* expression, and reduced tumor size and occurrence. The Axl tyrosine kinase receptor is associated with aggressive cancer and poor patient outcome in breast, liver and pancreatic cancer (Gjerdrum et al., 2010; Song et al., 2011; Reichl et al., 2015). Axl, and its γ-carboxylated ligand, Gas6, are associated with drug-resistant tumor relapse (Linger et al., 2008; Song et al., 2011; Schmidt et al., 2012; Kirane et al., 2015). We therefore evaluated novel combinations of standard-of-care PDA chemotherapeutics with Gas6/Axl signaling inhibitors in our rapid *in vivo* PDA therapeutic assay. We show that warfarin or an Axl kinase inhibitor (BGB324), in combination with gemcitabine and nab-paclitaxel, significantly reduce tumor initiation and growth. Thus, the *in vivo* PDA model harboring the Rgs16::GFP reporter is an efficient system for identifying effective drug combinations, and for identifying novel or repurposed drugs to treat PDA.

## RESULTS

### Rgs16::GFP is a Kras^G12D^-dependent reporter of PDA initiation and growth

We introduced the *Rgs16::GFP* reporter into *KC* and *KIC* mice because PDA initiates in pancreatic duct-like cells, either following ADM or neoplastic growth of progenitor cells, anywhere from the head to the tail of the pancreas but not in the hepatopancreatic duct (supplementary material Fig. S1) (Aguirre et al., 2003). The affected cell types are consistent with the expression domain of *p48* (*Ptf1a*) during pancreas development (Kawaguchi et al., 2002). Rgs16::GFP is expressed in embryonic and neonatal pancreatic ducts (Villasenor et al., 2010) and, in adults, in ducts early in the response to chronic high insulin demand and in mid-late gestation in pregnant females (Villasenor et al., 2010). Finally, endogenous *Rgs16* is expressed in human PDA [The Cancer Genome Atlas (TCGA), http://cancergenome.nih.gov/; Kim et al., 2010].

**Fig. 1.**
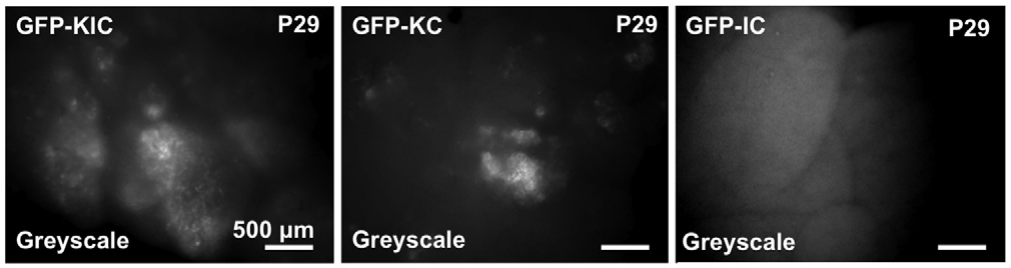
**Rgs16::GFP** **is a Kras^G12D^-dependent reporter of pancreatic neoplasia.** Rgs16::GFP is expressed in early pancreatic ductal neoplasia (IPMN, PanIN and PDA) but not in functional acinar cells of *Rgs16::GFP;KIC* (GFP-KIC) and *Rgs16::GFP; KC* (GFP-KC) mice (age=P29). No fluorescence background was detected in PDA tumors in mice lacking the *Rgs16::GFP* transgene (*KIC* or *KC* without GFP; not shown) nor in *GFP-IC* or normal glycemic *Rgs16::GFP* (GFP-WT; not shown) pancreata. Live fluorescence microscopy is shown. Scale bars: 1 mm.

**Fig. 2.**
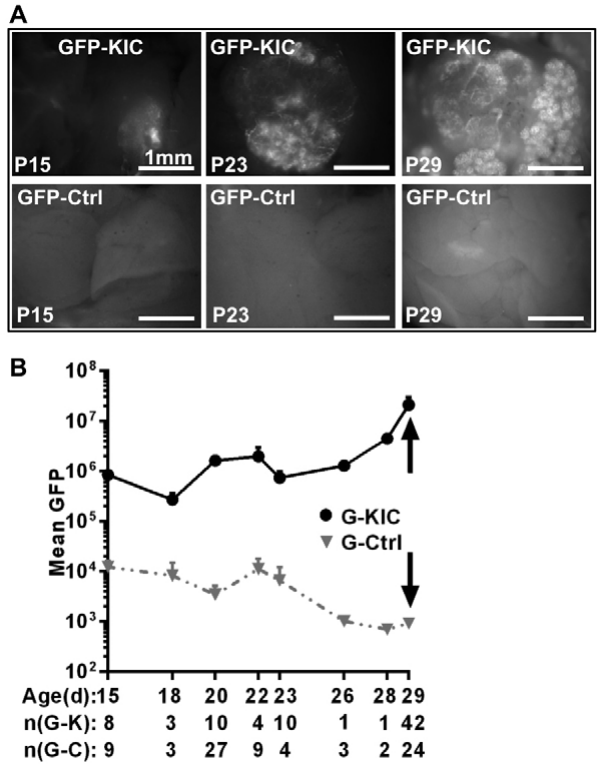
**Rgs16::GFP is a Kras^G12D^-dependent reporter of PDA expansion.** (A) Early lesions appear by postnatal day 15 (P15) in *Rgs16::GFP;KIC* (GFP-KIC) mice, marked by Rgs16::GFP expression in ducts. As tumors grow, GFP expression increases. (A; lower panels) Pancreata of normal glycemic, age-matched *Rgs16::GFP* (Ctrl) mice. Live fluorescence microscopy is shown. (B) Quantification of GFP expression based on the averages of the highest intensity pictures of each pancreas of *GFP-KIC* (G-KIC) and control (G-Ctrl) mice between P15 to P29. Rgs16::GFP expression increases with age and PDA expansion. GFP fluorescence of PDA tumors at P29 (arrows) is about 10,000-fold greater than non-tumorigenic, euglycemic *Rgs16::GFP* mice. Age and number of mice in *Rgs16::GFP;KIC* [n(G-K)] and *Rgs16::GFP*-Control [n(G-C)] groups are noted.

Analysis of pancreata harvested from postnatal day 29 (P29) mice demonstrated that Rgs16::GFP expression in pancreatic tumors is dependent on the (heterozygous) *Kras^G12D^* allele; *KIC* and *KC* mice had high Rgs16::GFP expression (Fig. 1). Pancreata in *KIC* mice at P29 had widespread ductal neoplasia. No GFP expression was observed in *IC* mice (Fig. 1), which do not harbor the *Kras^G12D^* allele and do not develop tumors. Background fluorescence was minimal in *KIC* mice lacking the GFP transgene (data not shown).

An important advantage of the *Rgs16::GFP;KIC* reporter mice is that the entire pancreas can be rapidly evaluated under a fluorescence dissection microscope to quantitate non-overlapping regions of GFP expression. In hundreds of pancreata dissected at multiple ages, every neoplastic lesion detected in bright-field microscopy expressed Rgs16::GFP (e.g. supplementary material Fig. S1A,B). A three-dimensional rotational movie shows Rgs16::GFP expression in a PDA tumor, several small areas of neoplasia and normal acinar cells in lobes at the head of the pancreas (supplementary material Movie 1; Fig. S2).

### Rgs16::GFP intensity increases with PDA initiation and growth

In *KIC* mice, Cre-recombinase is first expressed by the *p48* (*Ptf1a*) promoter in the embryonic progenitor cells that give rise to the three epithelial cell lineages in the adult – ducts, and exocrine and endocrine pancreas (Kawaguchi et al., 2002). Therefore, all cells in these pancreatic lineages express oncogenic *Kras^G12D^* and the tumor suppressor gene *Cdkn2a* is deleted (see supplementary material Fig. S3: the entire pancreas is marked by TdTomato in *p48^Cre^;LSL-TdT* reporter mice). Despite expression of *Kras^G12D^* and deletion of *Cdkn2a* throughout the pancreas, Rgs16::GFP is only expressed in a few early PanINs sparsely scattered throughout the pancreas 2 weeks after birth (P15; Fig. 2A). At P15, the pancreas appears morphologically and functionally normal, with the exception of these early PanINs. Thus, Rgs16::GFP reports the precise region of activated Kras^G12D^ signaling at tumor initiation and throughout progression.

Between the ages P15 to P29, average GFP fluorescence increased more than 100-fold as early PanINs appeared throughout the pancreas and PDA tumors grew (Fig. 2B). P29 is therefore an optimal time to assess tumor growth in weanlings because individual tumors achieve near-maximal brightness, more than 10,000-fold above background fluorescence.

### Rgs16::GFP is expressed in ADMs, PanINs and PDAs

**Fig. 3.**
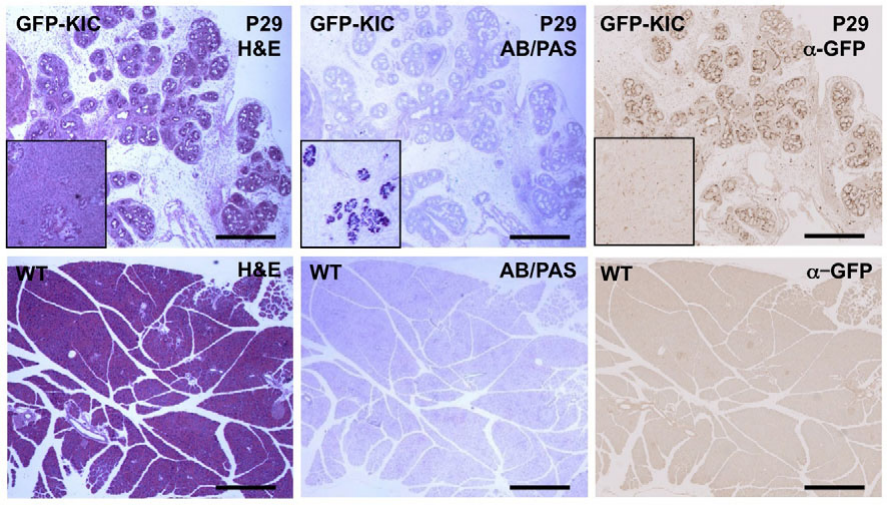
**Rgs16::GFP expression in pancreatic neoplasia.** Hematoxylin and eosin (H&E) staining for nuclear/cytoplasmic boundaries, Alcian Blue and Periodic Acid-Schiff (AB/PAS) staining for mucinous regions, and GFP staining for Rgs16 expression are shown in serial sections of *Rgs16::GFP;KIC* (GFP-KIC) pancreas. Inserts compare a representative region that is AB/PAS-positive but GFP-negative at low resolution. WT, wild type. Scale bars: 500 μm.

**Fig. 4.**
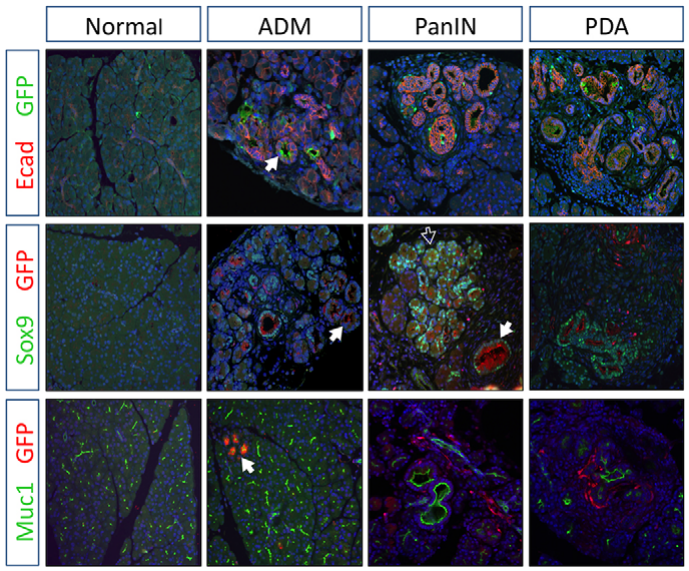
**Rgs16::GFP in ADMs, PanINs and PDAs.** Confocal images of immunofluorescence staining show Rgs16::GFP expression relative to Ecad (red), Sox9 (green) or Muc1 (green) in normal and neoplastic pancreas (ADM, PanIN, PDA) in *KIC* mice (P29). Primary features of particular interest (e.g. PanIN in the same field as ADM) are indicated by bold white arrows. Other features of interest (e.g. Sox9 expressed in ADM) are indicated by a white outlined arrow.

**Fig. 5.**
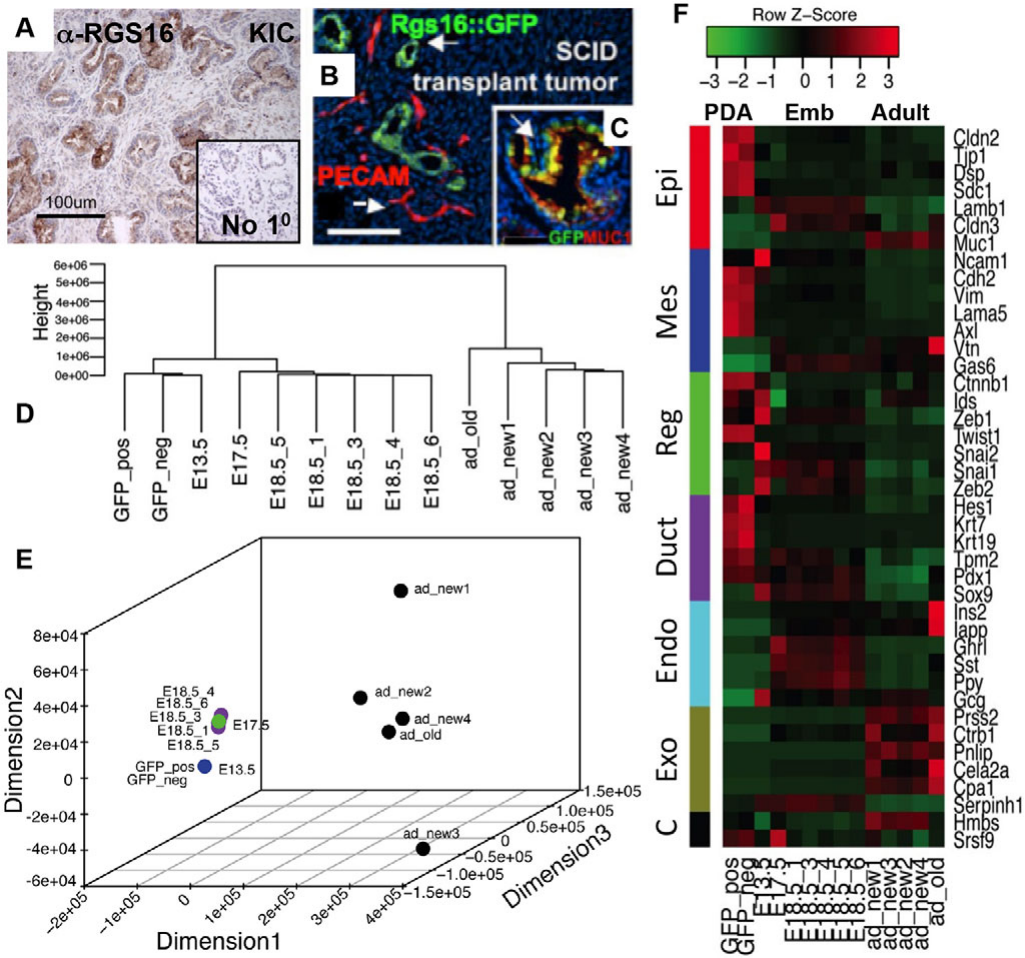
**Primary PDA cells express markers of pancreatic progenitor cells.** (A) Endogenous Rgs16 protein is expressed in ducts of primary tumors. Anti-Rgs16 immunohistochemical staining (brown) of a *KIC* mouse pancreas. The insert shows a section from the same tumor not stained with primary antibody. Background staining is Alcian Blue and Periodic Acid-Schiff. (B) Single cell suspension of PDA cells from tumor at 6 weeks transplanted orthotopically into a NOD-SCID recipient forms large tumors within 2 weeks. The close proximity of duct-like cancer (green; Rgs16::GFP) and vasculature (red; PECAM) is indicated by white arrows. Scale bar: 100 μm. (C) Primary tumor; Rgs16::GFP (green) and Muc-1 (red) are co-expressed (overlap appears yellow) in PanIN lesions of the ductal epithelium (white arrow). (D) Cluster dendrogram of RNA-Seq gene expression profiles. The dendrogram was obtained by hierarchical clustering of pairwise distances between all samples using normalized gene expression values. Each branch represents a sample: E18.5 and adult pancreas (five replicates each), PDA primary cell culture (cells sorted into Rgs16::GFP-positive and GFP-negative samples), E13.5 and E17.5 (one sample each). (E) 3D scatterplot showing dissimilarity between samples. We computed Euclidean distance between each pair of samples and scaled these distances using multidimensional scaling for representation in 3D space. Each axis represents a dimension and axis values represent range of dissimilarities between samples. (F) Heatmap showing the expression of markers in each sample. We selected a set of markers for developmental stages and computed the *z*-score to portray their relative expression levels in each sample. Red represents higher expression and green represents lower expression compared to population mean.

Pancreatic neoplasia secrete mucins and other polysaccharide-decorated proteins detected by Alcian Blue/Periodic Acid-Schiff (AB/PAS) staining. PDAs in *KIC* mice have intense Rgs16::GFP expression (supplementary material Fig. S4) but little or no AB/PAS staining, whereas Rgs16::GFP is significantly lower in regions of neoplasia that are AB/PAS-positive (Fig. 3, inserts). High-resolution confocal microscopy revealed that Rgs16::GFP is not expressed in normal acinar cells, consistent with fluorescence microscopy of dissected pancreata (Figs 1,2). Kras^G12D^-evoked ADM induced *Sox9* expression, as previously reported (Kopp et al., 2012; Krah et al., 2015), and low Rgs16::GFP expression was observed in most cells (Fig. 4). By contrast, Rgs16::GFP expression was significantly higher and co-expressed with Sox9 in duct-like PanIN lesions, consistent with the findings shown in Fig. 3. The marker of proliferation-competent cells, Ki67, was co-expressed with Rgs16::GFP in many cells within PDAs and PanINs but almost never in regions of ADM (supplementary material Fig. S5). This pattern of high Rgs16::GFP expression in PanINs and PDAs was confirmed by immunofluorescence with Muc1, Ecad and endogenous Rgs16 (Fig. 4, Fig. 5A). Exceptional cases induced intense Rgs16::GFP expression in acinar-like cells (supplementary material Fig. S6) co-expressing high carboxypeptidase A1 (CPA1; supplementary material Fig. S7). This pattern was observed in peripheral lobes with edema that sit beyond (proximal to) tumor nodules in *KIC* pancreata. These seem to be specialized responses in which Rgs16 and CPA1 expression is secondary to PDA tumor growth.

### Rgs16::GFP is expressed in pancreatic ductal progenitor and PDA cells

*KIC* mice (6-8 weeks) and PDA primary cells in culture co-expressed Rgs16::GFP with markers of pancreatic progenitor cells and epithelial-mesenchymal transition (EMT), such as Sox9, Muc1 and Ecad (Fig. 4, Fig. 5C; β-catenin and TGF-β supplementary material Fig. S8). Orthotopic transplantation of Rgs16::GFP^+^ primary cells derived from PDA tumors at 6 weeks rapidly regenerated GFP-positive pancreatic cancer in duct-like structures in close proximity with vasculature in recipient NOD-SCID mice (Fig. 5B). By contrast, stroma of the non-transgenic host did not express GFP.

To gain more information about the gene expression profile of the *KIC* PDA cells, we performed RNA sequencing (RNA-Seq) analysis of the transcriptome, and found that PDA cells in primary culture are closely related to embryonic (E13.5) pancreas (Fig. 5D,E). PDA cells and E13.5 progenitor cells of the ductal and endocrine lineages expressed Pdx-1 and Sox9 (Fig. 5F). Similarities in the transcriptomes diverged noticeably by E17.5 and E18.5, as pancreata begin to express more genes in maturing endocrine and exocrine cells, and fewer mesenchymal genes. The transcriptomes of PDA primary cells and normal adult pancreas are only distantly related; PDA cells did not express appreciable levels of markers of mature acinar or endocrine cell types.

In a survey of cancer-associated receptors and ligands in primary PDA cells in culture, we noted that the receptor tyrosine kinase Axl was highly expressed, with modest expression of its endogenous ligand Gas6 (Fig. 5F, Table 1). Axl and Gas6 are highly expressed in many human primary PDA tumors (TCGA data), containing both cancer and stromal cells. Axl was the most highly enriched receptor kinase expressed in PDA cells for which we had inhibitors of active ligand maturation and receptor antagonists to test PDA initiation and progression. Furthermore, Axl is associated with EMT and drug resistance in carcinomas (Zhang et al., 2012; Byers et al., 2013). Therefore, we sought to establish a rapid *in vivo* assay to assess the effect of clinical Axl inhibitors on PDA initiation and progression.

**Table 1.**
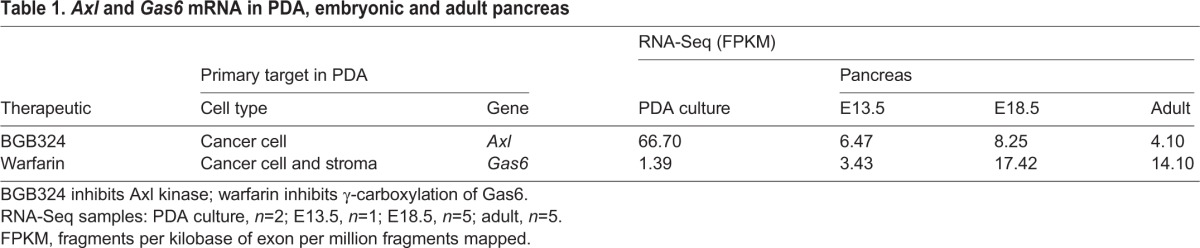
***Axl* and *Gas6* mRNA in PDA, embryonic and adult pancreas**

### A 2-week *in vivo* assay: PanIN and PDA tumor growth is suppressed at P29 by a combination of Axl inhibitors, gemcitabine and Abraxane

The therapeutic screening strategy was to treat *KIC* pups between P15-P28 (when PanINs and PDAs are in rapid expansion) with anticancer drugs, sacrifice mice at P29, and capture Rgs16::GFP intensity with a dissection fluorescence microscope. Images of the five brightest non-overlapping fields of Rgs16::GFP expression were then collected, representing the regions of greatest tumor burden (supplementary material Fig. S9). This quantitative protocol represents a much more rapid approach (about 5% of the time) than traditional histology for analysis of the entire pancreas from each mouse in a cohort of 20 adults.

**Fig. 6.**
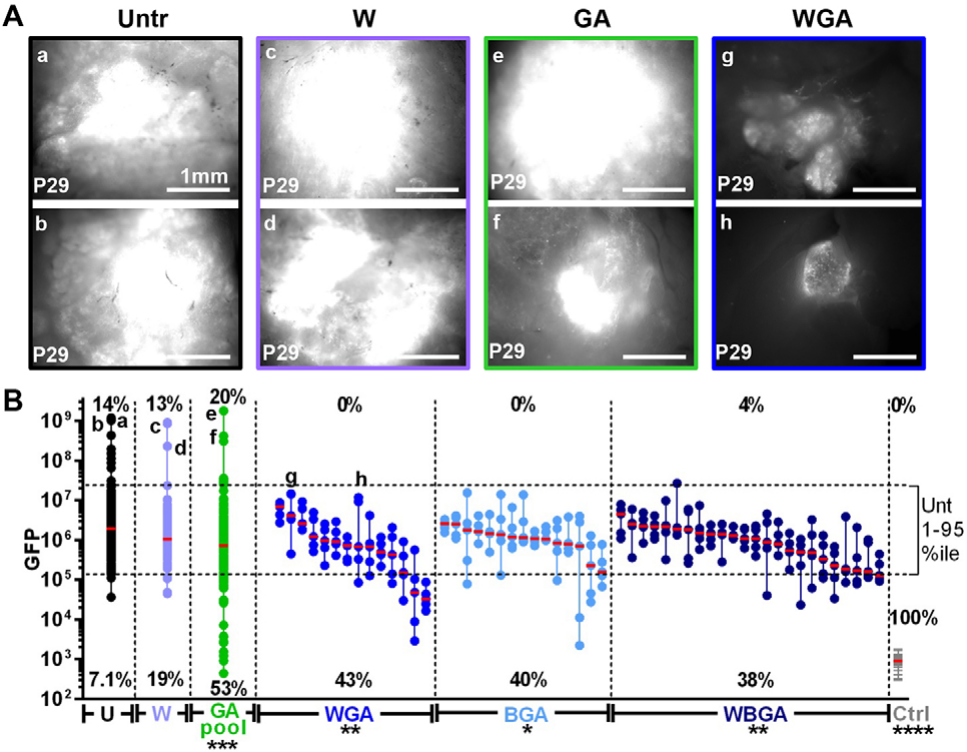
**A 2-week *in vivo* assay: PDA tumor growth and PanINs suppressed at P29 by Axl inhibitors+gemcitabine+Abraxane.** (A) The two highest intensity images of *Rgs16::GFP;KIC* mice at P29 are shown for the (a,b) untreated (Untr), (c,d) warfarin (W), (e,f) gemcitabine and Abraxane (GA), and (g,h) warfarin with gemcitabine and Abraxane (WGA) groups. (B) Gemcitabine+Abraxane (GA) with the Gas6/Axl inhibitors warfarin (dark blue dots; WGA, *n*=14), BGB324 (light blue dots; BGA, *n*=15) or both (WBGA, black dots on the right, *n*=24) inhibit growth of large PDAs and reduce PanINs throughout the pancreas (many fields with little or no GFP; *n*=53). GA alone (green dots, *n*=30) reduces PanINs but resistant tumors occur (many fields with high GFP). Warfarin alone (W, pale blue dots on the left, *n*=16) has no effect, similar to untreated control (U, black dots on the left, *n*=42). WGA, BGA and WBGA show pancreata from individual mice; each column of five dots represents one pancreas, each dot a single micrograph, non-overlapping fields from 1st to 5th brightest (quantitated by ImageJ; statistics in supplementary material Table S1). All of the untreated (U), gemcitabine+Abraxane (GA), and warfarin (W) group images are collapsed into a single vertical alignment each to save space. The response to gemcitabine alone (*n*=30) and gemcitabine+warfarin (*n*=33) is shown in supplementary material Fig. S10; statistics in supplementary material Table S2. Control mice (Ctrl, *Rgs16::GFP* transgenics, gray lines, *n*=24) are represented as one line per mouse. The 95-percentile and 1-percentile of all image values within untreated group are depicted with dashed horizontal lines and the percentage of mice with images above and below, respectively, of these values are indicated for each group. Small alphabetical letters correspond to images in panel A. Mean log (GFP) of each group is shown (supplementary material Table S1). Pancreata of untreated PDA mice express significantly more Rgs16::GFP than treated groups (GA, BGA, WGA). Student's *t*-test, *P<0.05, **P<0.01, ***P<0.001, ****P<0.0001.

To validate this *in vivo* screening approach, we treated *KIC* mice with gemcitabine+Abraxane (GA), a current standard-of-care for PDA therapy (Von Hoff et al., 2013). GA therapy significantly reduced PanIN lesions throughout the pancreas (supplementary material Fig. S9B; note the fields of low GFP expression in treated animals compared to untreated animals), and reduced average and median GFP expression (Fig. 6B). GA was more effective than gemcitabine alone at inhibiting PanIN initiation, relative to untreated controls (supplementary material Figs S9, S10, statistical analysis in supplementary material Tables S1, S2). Thus, this method detected that a standard-of-care drug treatment was able to impede tumor growth. However, toxic side effects inhibited growth of weanling mice (supplementary material Fig. S11). Furthermore, although GA treatment reduced PanIN lesions, large PDA tumors emerged at the same frequency in untreated and GA-treated mice (three trials), consistent with its modest effects in humans (Becker et al., 2014). As in humans, tumor heterogeneity exists between mice, reflected by differences in Rgs16::GFP expression levels, histology, immunofluorescence and response to treatment in *KIC* mice. Heterogeneity also exists within each pancreas – some regions were apparently unaffected at P29 whereas other areas had ADM, PanIN, and/or small or large PDA tumors.

To address whether blocking Axl signaling would improve GA effectiveness, we tested two clinical Axl inhibitors, BGB324 (Ben-Batalla et al., 2013) and warfarin, in combination with GA in PDA primary cell culture and *KIC Rgs16::GFP* reporter mice. In PDA primary cell culture, high concentrations of gemcitabine killed cells, and BGB324 was cytostatic, but warfarin had no effect (supplementary material Fig. S12). In *KIC* mice, warfarin had no effect by itself but, when combined with gemcitabine+Abraxane (WGA), PanIN lesions were further reduced and, importantly, WGA significantly reduced the growth of large PDA tumors observed in untreated and GA-treated mice (Fig. 6). Similar inhibitory effects were seen when GA was combined with BGB324 (BGA) or warfarin plus BGB324 (WBGA) (Fig. 6). Together, these data demonstrate that this screening strategy in *KIC Rgs16::GFP* mice is a relatively rapid means for identifying novel or repurposed drugs, as well as new drug combinations, for treatment of Kras-dependent PDA.

## DISCUSSION

The dismal survival rate of patients diagnosed with pancreatic cancer justifies an intense preclinical effort to identify novel PDA therapeutics. GEMMs have substantial benefits for drug screening, including recapitulation of all major stages of PDA and the complex interplay between precancerous neoplasia, adenocarcinoma, immune components and stromal elements (Cook et al., 2012). We used the *KIC* model because it is currently the most aggressive GEMM of PDA available (Aguirre et al., 2003). *KIC* mice harbor the two earliest genetic mutations common to most individuals with PDA: oncogenic mutations in *Kras*, which confer GAP-resistance (e.g. *Kras^G12D^*), and deletions of the tumor suppressor *Cdkn2a*. In the *KIC* GEMM, neoplasia initiate around 2 weeks of age and tumors (1-2 mm) develop in all untreated mice by P29. Furthermore, toxic and off-target drug effects inhibit growth of weanling mice and are easily measured as reduced body weight (supplementary material Fig. S11). Although weanlings might be hypersensitive to toxic drugs, the screen allows a rapid assessment of *in vivo* drug efficacy and specificity in weanling pups at a relatively modest cost.

To accelerate the discovery of effective drugs that inhibit PDA *in vivo*, we developed Rgs16::GFP as a sensitive reporter of PanIN and PDA initiation, progression and tumor size by 4 weeks of age (P29) in *KIC* mice. Rgs16::GFP expression is Kras^G12D^-dependent in *KC* and *KIC* mice. GFP is not expressed in *IC* mice (which do not develop neoplasia), and PDAs that develop in the GEMMs have no background fluorescence in the absence of the GFP transgene. Endogenous Rgs16 is not abundant in normal adult exocrine pancreas, nor is it usually induced in dedifferentiating (injured) acinar cells early in the process. For example, *Rgs16* is not induced in the pancreas (primarily acinar cells) of adult *Ptf1a* knockout (KO) mice, although CPA1 (an acinar cell marker) declines precipitously during the 2-week progression of acinar cell de-differentiation (Krah et al., 2015). Kras^G12D^ opposes Ptf1a maintenance of terminally differentiated pancreas and promotes acinar dedifferentiation in P29 *KC* and *KIC* mice (Krah et al., 2015). Widespread, high Rgs16::GFP expression in acinar-like cells (co-expressing high CPA1; supplementary material Fig. S7) was sometimes observed in peripheral lobes with edema that sit beyond (proximal to) tumor nodules in *KIC* pancreata (supplementary material Fig. S6). These are specialized responses in which *Rgs16* and *CPA1* expression is secondary to PDA tumor growth. Tumors and/or calcified ductal stones might block the duct [analogous to partial pancreatic ductal ligation (Xu et al., 2008)], and promote edema and signaling that stimulates this unusually intense co-expression of *CPA1* and *Rgs16*. Whereas *Rgs16* expression persisted, co-expression with *CPA1* in these dedifferentiating pancreatic lobes was presumably transient, because these structures (and CPA1) were absent in PanIN and in older *KIC* mice with solid tumors occupying the entire transformed pancreas.

An important point is that, although all pancreatic cells in *KIC* mice express Kras^G12D^ and have inactivated Cdkn2a (indeed, *p48::Cre* drove TdTomato expression throughout the pancreas; supplementary material Fig. S3), Rgs16::GFP is absent at P15 except for expression in the earliest lesions, and then throughout tumor progression. Huang et al. (2014) showed that oncogenic Kras requires GTP loading for enhanced activity. Presumably, Rgs16::GFP is marking the precise location of, and perhaps the cells directly engaged in, receptor-dependent activation of Kras^G12D^ signaling. Thus, these *in vivo* reporter mice could help to identify drugs that directly or indirectly inhibit Kras^G12D^ activation during ADM, PanIN formation and PDA progression.

We used Rgs16::GFP to evaluate novel PDA therapeutic combinations in a rapid (2 week) *in vivo* screen. GFP intensity increased as a function of tumor burden in the pancreas (Fig. 2). Therefore, drugs and novel small molecules that inhibit PDA progression *in vivo* can be readily identified by their ability to reduce Rgs16::GFP fluorescence intensity in dissected pancreata (Fig. 6, supplementary material Figs S9, S10). We showed that a standard-of-care combination of gemcitabine+Abraxane (GA) reduced initiation of neoplasia in *KIC;Rgs16::GFP* reporter mice. However, GA did not significantly reduce growth of the largest pancreatic tumors, consistent with modest effects in prolonging survival in humans (Becker et al., 2014).

To identify additional genes that might be involved in PDA initiation and tumor growth, and therefore be potential targets for drug treatment, we characterized the gene expression profile of primary PDA cells in culture by RNA-Seq. The transcription profile of PDA cells was most closely related to embryonic progenitors of ducts and islets, the same embryonic cell types that express Rgs16 (Villasenor et al., 2010). Rgs16::GFP is a marker of embryonic pancreatic stem cells at E9.0 in the pancreatic bud, and continues to be expressed in Sox9-positive duct cells and insulin-positive endocrine cells at E15.5 (Villasenor et al., 2010). Thus, monitoring Rgs16 expression might lead to the identification of receptors and ligands important in stem cell function and cancer initiation. We found that expression of the tyrosine kinase receptor Axl was tenfold higher in primary PDA cells compared to normal embryonic (E13.5) pancreas and 16-fold higher than normal adult pancreas (Fig. 5F; Table 1), consistent with observations by others (Song et al., 2011). It was found that *Axl* and *Rgs16* tend to be coordinately expressed in human PDA primary tumors (from analysis of co-expression of the human primary PDA samples characterized in supplementary material Figs S13 and S14). High levels of *Axl* expression in advanced cancers from diverse cellular origins suggest that tumor-cell-associated Axl might be a fundamental contributor to malignant progression (Holland et al., 2010). Indeed, our recent results support the notion that Axl signal transduction is required to maintain epithelial plasticity traits of aggressive pancreatic tumors, including tumorigenicity, invasiveness, survival, drug sensitivity and metastasis (Kirane et al., 2015). Additionally, Axl inhibition has been shown to block cell migration and reduce metastasis in breast cancer models (Gjerdrum et al., 2010; Paccez et al., 2013; Dunne et al., 2014; Paolino et al., 2014).

We found that Gas6, Axl ligand, was expressed in normal adult pancreas (and primary human PDA tumors; TCGA) but its expression was tenfold lower in PDA primary culture cells (Table 1). Warfarin inhibits post-translational γ-carboxylation of Gas6, which is necessary for its ability to activate Axl signaling (Lew et al., 2014). Whereas warfarin by itself had no effect on PDA, warfarin combined with gemcitabine+Abraxane (WGA) had three important effects in *KIC;Rgs16::GFP* reporter mice. Compared to GA therapy alone, WGA further reduced the initiation of neoplasia, lowered median tumor size, and significantly reduced growth of the largest tumors. We used low doses of WGA that have, individually, proven safe in humans. WGA retarded growth in weanling mice but this is attributable to gemcitabine and Abraxane. We have recently shown that warfarin exerts its anti-cancer effects by inhibiting Gas6-mediated Axl activation in PDA tumor cells (Kirane et al., 2015). A selective Axl kinase inhibitor, BGB324, had similar effects on PDA initiation and growth when combined with GA (manuscript in preparation). Systemic Axl inhibition might also exert anti-tumor effects through host-response-dependent mechanisms (Paolino et al., 2014; Kirane et al., 2015). We screened a total of 53 mice with GA plus warfarin or BGB324. All mice survived treatment from P15 to P29, and about 40% had lower rates of neoplasia compared to the control mice that we analyzed.

Rgs16::GFP expression is essentially extinguished in pancreas of normal, euglycemic mice by P15 and completely absent by P28 (Villasenor et al., 2010). By contrast, Rgs16::GFP expression in the largest PDA tumors was about 1-million times brighter than control pancreas, or regions of *KIC* pancreas not yet affected by Kras^G12D^ expression. Equally important for identification of the most effective PDA therapeutics, the median GFP expression in untreated mice was 1000-fold higher than non-GFP *KIC* mice, or *Rgs16::GFP* control mice lacking PDA. Warfarin+GA significantly retarded PDA initiation and progression. Although this *in vivo* assay is sensitive and rapid, it is primarily a chemopreventive screen in young animals, whereas PDA typically initiates in middle age and is diagnosed late in life. Therefore, the best drug candidates identified in this rapid *in vivo* assay should be validated in survival and tumor regression studies in adults. An early phase clinical trial is under consideration for low-dose warfarin based on the combination of findings we report here, other preclinical studies (Brown, 1973; McCulloch and George, 1987; Schulman and Lindmarker, 2000; Kirane et al., 2015) and anecdotal observations in patients (Brown, 1973; McCulloch and George, 1987; Schulman and Lindmarker, 2000; Kirane et al., 2015). Although we still found significant tumor progression in WGA-treated *KIC* mice, this might be caused by other receptors activating Kras^G12D^. Further inhibition might be achieved by adding another inhibitor to the combination therapy.

In summary, the screening method described here reveals sensitivity to new drug regimens that inhibit Kras^G12D^-mediated oncogenesis. These findings suggest that patients with successful resection of PDA and clear margins of resection might benefit most from repurposed low-dose warfarin treatment in combination with gemcitabine chemotherapy. Future studies will test new drugs as they become available to help identify the most effective and targeted PDA therapeutics.

## MATERIALS AND METHODS

### Mouse lines and genotyping

The mouse lines used were *KIC* (*p48^Cre/+^; Kras^G12D/+^; Cdkn2a^f/f^*); *KC* (*p48^Cre/+^; Kras^G12D/+^*); *IC* (*p48^Cre/+^; Cdkn2a^f/f^*); *Rgs16::GFP;* *KIC* [*KIC* mice crossed with *Rgs16::GFP* BAC transgenic mice to generate *Rgs16::GFP;p48^Cre/+^;Kras^G12D/+^;Cdkn2a^f/f^* (*Rgs16::GFP;KIC*) reporter mice]. Genotyping was done using clipped tails before sacrifice and confirmed with spleen DNA after dissection. Mice were maintained at a 12-h day, 12-h night cycle on normal chow *ad libitum* according to the rules and standards of UT Southwestern Institutional Animal Care and Use Committee. *Rgs16* mice were identified by blue light excitation of GFP in the brain of newborn pups or in the retina of adult mice. Genotyping of *KIC* mice was done with the following primers: p48^Cre^ (For: 5′-CCTGGAAAATGCTTCTGTCCG-3′; Rev: 5′-CAGGGTGTTATAAGCAATCCC-3′; product: 392 bp), LSL-Kras^G12D^ (For: 5′-CTAGCCACCATGGCTTGAGT-3′; Rev: 5′-TCCGAATTCAGTGACTACAGATG-3′; product: 327 bp) and Cdkn2a^f/f^ (For: 5′-TTGTTGGCCCAGGATGCCGACATC-3′; Rev: 5′-CCAAGTGTGCAAACCCAGGCTCC-3′; product: 145 bp for wild type, 179 bp for *loxP* inserted allele). All PCR conditions started with genomic denaturation at 94°C for 10 min followed by 33 cycles of 94°C denaturation for 30 s, 60°C annealing for 1 min, and 72°C elongation for 1 min. PCR products were run in a 1% agarose gel.

### Fluorescent microscopy and GFP quantification

Pancreatic expression of Rgs16::GFP in *Rgs16::GFP;KIC* mice was captured under a Zeiss Lumar tissue dissection microscope (eye piece =10×) with Filter Set Lumar 13 (excitation: BP 470/20, emission: BP 505-530) illuminated by Osram HBO 103 W/2 mercury short-arc (without reflector) fluorescent lamp. The microscope objective was NeoLumar S (0.8×, FWD 80 mm) and the total image magnification was selected to be 25.5×. Images were captured via a single-channel camera (Hamamatsu 60-C, 1″, 1×) in 1344×1024 resolution with 1 s exposure and 1×1 binning, analog gain=10, and analog offset=2 settings. Pancreatic fields representing the tumor burden (three to four fields for P15 pups and five or more for pups from P23 onwards) of the pancreas were imaged, covering up to 50% of the organ surface area. All images were saved in gray-scale 16-bit tiff format. Images were quantified using NIH ImageJ software with background subtraction with a radius of 50 pixels. A variable and tight threshold was set to eliminate residual background. Intensities of all particles with size ≥5 pixels were summed to obtain the total light intensity per image.

### Tissue clearing and microscopy

Pancreata and a small section of duodenum containing the ampulla of Vater were dissected, fixed overnight in PFA, and washed in PBS (4°C). Tissues were stored in PBS (4°C). Tissue clearing and microscopy was done as described by Soderblom et al. (2015), based on previous studies (Becker et al., 2012; Kopp et al., 2012; Krah et al., 2015). Production of the three-dimensional (3D) rotational movie was done as described by Soderblom et al. (2015) using IMARIS.

### Drug dosages

*Rgs16::GFP;KIC* mice were injected intraperitoneally with gemcitabine (Eli Lilly; Indianapolis, IN, USA) (12.5 mg/kg body weight/day given 3 days/week, dissolved in PBS), Abraxane (Cellgene; Summit, NJ, USA) (5 mg/kg body weight/day given 2 days/week, diluted in 2% saline solution), BGB324 (BerGenBio, Bergen, Norway) (5 mg/kg body weight/day given 5 days/week, dissolved in a mix of DMSO:ethanol:Kolliphor EL:water with 4:4:8:84 v/v ratio), and warfarin (0.2 mg/kg body weight/day given 5 days/week, dissolved in PBS) according to the injection schedules specified in supplementary material Fig. S9. These doses were validated in previous mouse studies (Dineen et al., 2010; Kutluk Cenik et al., 2013; Ostapoff et al., 2013, 2014; Aguilera et al., 2014). Note that 30% cyclodextrin was discontinued as a vehicle for BGB324 (v2B, v2G) because it was not active. GA was not affected (supplementary material Fig. S9; GA_v2B, GA_v2G). To verify that tumor progression remains constant over the duration of these experiments, assays of untreated mice were interspersed with drug-treated cohorts, and warfarin-treated pups were the final cohort we tested. Gemcitabine, Abraxane and warfarin were purchased from the UT Southwestern clinical pharmacy; BGB324 was a gift from BerGenBio.

### *Rgs16::GFP* PDA primary cell culture

Primary *Rgs16::GFP* PDA cells were harvested from 6-week-old *KIC* mice. Cells were grown in 25 mM Glucose DMEM (HyClone) with 10% FBS (Serum Source International), penicillin and streptomycin (Life Technologies) on rat tail collagen type 1 (BD Biosciences) coated plates (0.5 µg/cm^2^) in a humidified incubator at 37°C and 5% CO_2_. Cells reaching confluency were washed twice with PBS (HyClone) and lifted with 0.05% Trypsin-EDTA (HyClone) treatment up to 10 min in the incubator. For drug tests in supplementary material Fig. S12, primary PDA cells were incubated with gemcitabine (10 μM dissolved in PBS), warfarin (1 μM or 10 μM dissolved in water), BGB324 (1 μM or 10 μM dissolved in DMSO) or warfarin and BGB324 together for 24 h in a 37°C incubator. PDA live cell numbers were obtained via counts using a hemocytometer following 2% trypan blue staining to distinguish dead cells under an inverted microscope.

### PDA primary cell RNA-Seq

Cultured *Rgs16::GFP* PDA cells were stimulated with 40% FBS containing growth medium, incubated overnight and subjected to FACS to separate the GFP-positive and -negative cell populations. After isolating RNA via TRIzol (Life Technologies) treatment, the transcriptional profile of each GFP-PDA population was revealed via RNA-Seq performed on poly-A selected mRNA. Mouse sequence reads were aligned to the mm9 genome assembly using TopHat v2.0.9 (Trapnell et al., 2009). All default settings were used except: -G option and -no-novel-juncs. The Cuffdiff module available in Cufflinks software v2.1.1 was used to quantify the expression by the FPKM method (Trapnell et al., 2010, 2012). The geometric method (median of the geometric means of fragment counts across all libraries) was used to normalize and scale FPKM values.

### Dendrogram – mouse PDA culture and tissues

We calculated pair-wise distances between all array sample expression data using the ‘euclidean’ method in dist () R function to check the similarities between samples. This method calculates the distance between the two vectors. We performed hierarchical clustering on this distance matrix using the ‘ward’ method in hclust () R function.

### Dendrogram – TCGA RNA-Seq

We extracted pancreatic adenocarcinoma patient sample (*n*=178) mRNA expression data available from The Cancer Genome Atlas (TCGA). These data contain normalized gene expression in terms of transcripts per million (TPM) and these values were used for the further analysis. To compare expression levels in the human PDA tumor samples with mouse samples, we used the NCBI homologene database to extract mouse homolog genes for human genes. From this list, we selected genes that show ≥10 TPM in at least 10% of the human primary tumor samples. This filter retained 10,135 genes used to plot the dendrogram.

### Multidimensional scaling

We used multidimensional scaling (MDS) to assess the differences between samples. For this, we used normalized expression matrix for all genes in the genome across all samples and computed distances between each sample pair using the euclidean method; cmdscale () in stats R package (R Development Core Team, 2014) was used to represent these distances between each pair of samples in 3D space.

### Heatmap

Z-score was computed for a selected set of markers in each category across all samples and plotted using heatmap.2 () function available in gplots R package (Warnes et al., 2015).

### Statistical analysis of GFP expression

GFP values were converted to log_10_ scale prior to statistical analysis. Graphs and their statistical comparisons were done using GraphPad Prism software with unpaired and two-tailed Student's *t*-test. Significance between groups was indicated as ns (not significant); *P<0.05; **P<0.01; ***P<0.001; ****P<0.0001. Error bars in all the graphs are standard error of the mean (s.e.m.). See supplementary material Tables S1 and S2 for additional statistical analysis.

## Notes

This article is part of a subject collection on Model Systems in Drug Discovery: from Bench to Patient. See related articles in this collection at http://dmm.biologists.org/cgi/collection/drugdiscovery
